# Human decision making anticipates future performance in motor learning

**DOI:** 10.1371/journal.pcbi.1007632

**Published:** 2020-02-28

**Authors:** Joshua B. Moskowitz, Daniel J. Gale, Jason P. Gallivan, Daniel M. Wolpert, J. Randall Flanagan

**Affiliations:** 1 Centre for Neuroscience Studies, Queen’s University, Kingston, Ontario, Canada; 2 Department of Psychology, Queen’s University, Kingston, Ontario, Canada; 3 Department of Biomedical and Molecular Sciences, Queen’s University, Kingston, Ontario, Canada; 4 Department of Engineering, University of Cambridge, Cambridge, United Kingdom; 5 Department of Neuroscience, Mortimer B. Zuckerman Mind Brain Behavior Institute, Columbia University, New York, New York, United States of America; Johns Hopkins University, UNITED STATES

## Abstract

It is well-established that people can factor into account the distribution of their errors in motor performance so as to optimize reward. Here we asked whether, in the context of motor learning where errors decrease across trials, people take into account their future, improved performance so as to make optimal decisions to maximize reward. One group of participants performed a virtual throwing task in which, periodically, they were given the opportunity to select from a set of smaller targets of increasing value. A second group of participants performed a reaching task under a visuomotor rotation in which, after performing a initial set of trials, they selected a reward structure (ratio of points for target hits and misses) for different exploitation horizons (i.e., numbers of trials they might be asked to perform). Because movement errors decreased exponentially across trials in both learning tasks, optimal target selection (task 1) and optimal reward structure selection (task 2) required taking into account future performance. The results from both tasks indicate that people anticipate their future motor performance so as to make decisions that will improve their expected future reward.

## Author summary

A hallmark of motor learning is the reduction of performance errors with practice, which can have important ramifications for decision making. For example, when purchasing a new surfboard, our choice should anticipate our improvement with practice so that we select an appropriate board. In this paper we asked whether, in the context of two different motor learning tasks, people take into account their future, improved performance so as to make optimal decisions to maximize reward. In the throwing task, which involved learning the dynamics of the thrown object, participants were periodically given the opportunity to select from a set of smaller targets of increasing value. In the reaching task, which involved adapting to a visuomotor rotation, participants—after performing an initial block of trials—selected a reward structure (ratio of points for target hits and misses) for different exploitation horizons (i.e., numbers of trials they might be asked to perform). Performance errors decreased across trials in both tasks and therefore optimal target selection (task 1) and optimal reward structure selection (task 2) required taking into account future performance. In both tasks, participants anticipated their future motor performance so as to make decisions that improved their expected future reward.

## Introduction

An inherent component of sensorimotor control involves dealing with movement variability that arises from sensory and motor noise [1–3]. It has been shown, in the context of both eye and arm movements, that motor planning takes into account signal-dependent noise in motor commands so as to minimize endpoint variance [4]. Moreover, in line with current theories of feedback control [5–7], it has been shown that on-line corrections for errors, arising from noise or perturbations, minimize task-relevant variability that threatens the successful completion of the goal. For example, when responding to errors in lateral hand position during reaching, the gain of the corrective response is greater when the target is narrow than when it is wide [8,9].

Studies examining reward optimization during target-directed reaching have shown that people can accurately estimate their motor variability [e.g., 10,11–13]. In a task developed by Trommershäuser and colleagues [14], participants made rapid pointing movements towards partially overlapping reward and penalty regions (circles) presented on a computer touchscreen. These studies showed that participants selected an aim location (which could be far from the center of the reward region) that was near-optimal in terms of maximizing reward, indicating that they accurately incorporated their own movement variability in motor planning [14,15]. A subsequent experiment showed that when presented with two configurations, each consisting of a reward region and a partially overlapping penalty region, participants rapidly selected, and then optimally aimed towards, the configuration that had the highest expected gain given their movement variability [16]. These results suggest that people can rapidly take into account their movement variability when making strategic decisions about where to reach. Interestingly, when movement variability was artificially increased via altered visual feedback, participants accurately updated their estimate of this variability so as to achieve near-optimal performance [17–19].

In the main studies described above, a well practiced movement—i.e., simple target directed reaching—was employed such that the distribution of errors about the desired aim point is fixed. However, in many motor learning tasks, errors typically decrease across trials through a combination of reduction in bias and variance [20,21]. For example, when adapting to a visuomotor rotation, which rotates the viewed position of the hand about that movement start point, reduction in error largely involves reducing the bias in reach direction caused by the rotation. In contrast, in many skill learning tasks, reduction of error primarily involves reducing the variance. This reduction in error associated with motor learning can have important ramifications for decision making [22]. For example, as a squash player’s skill improves, they will want to transition from the bounciest entry level ball (blue dot) through to less bouncy balls (red, yellow and ultimately double yellow dot). In choosing a ball for a match, a novice might want to consider their likely improvement during the match when selecting a ball. For example, if they predict that they will improve they may go for a red or even yellow dot ball as opposed to the blue dot ball they may have used last time out. Similarly, when renting or purchasing a pair of skis, a surfboard, or a tennis racquet, our choice should anticipate our likely improvement over time through motor learning. More generally, there are many situations that involve a commitment—such as selecting the difficulty of courses at university—in which optimal decisions need to consider expected learning.

The aim of the current study was to test the hypothesis that people can optimize reward during motor learning by taking account of their current and, critically, their future motor performance. We tested this hypothesis using two different motor tasks in which predictions about future performance were evaluated in different ways.

**Fig 1:**
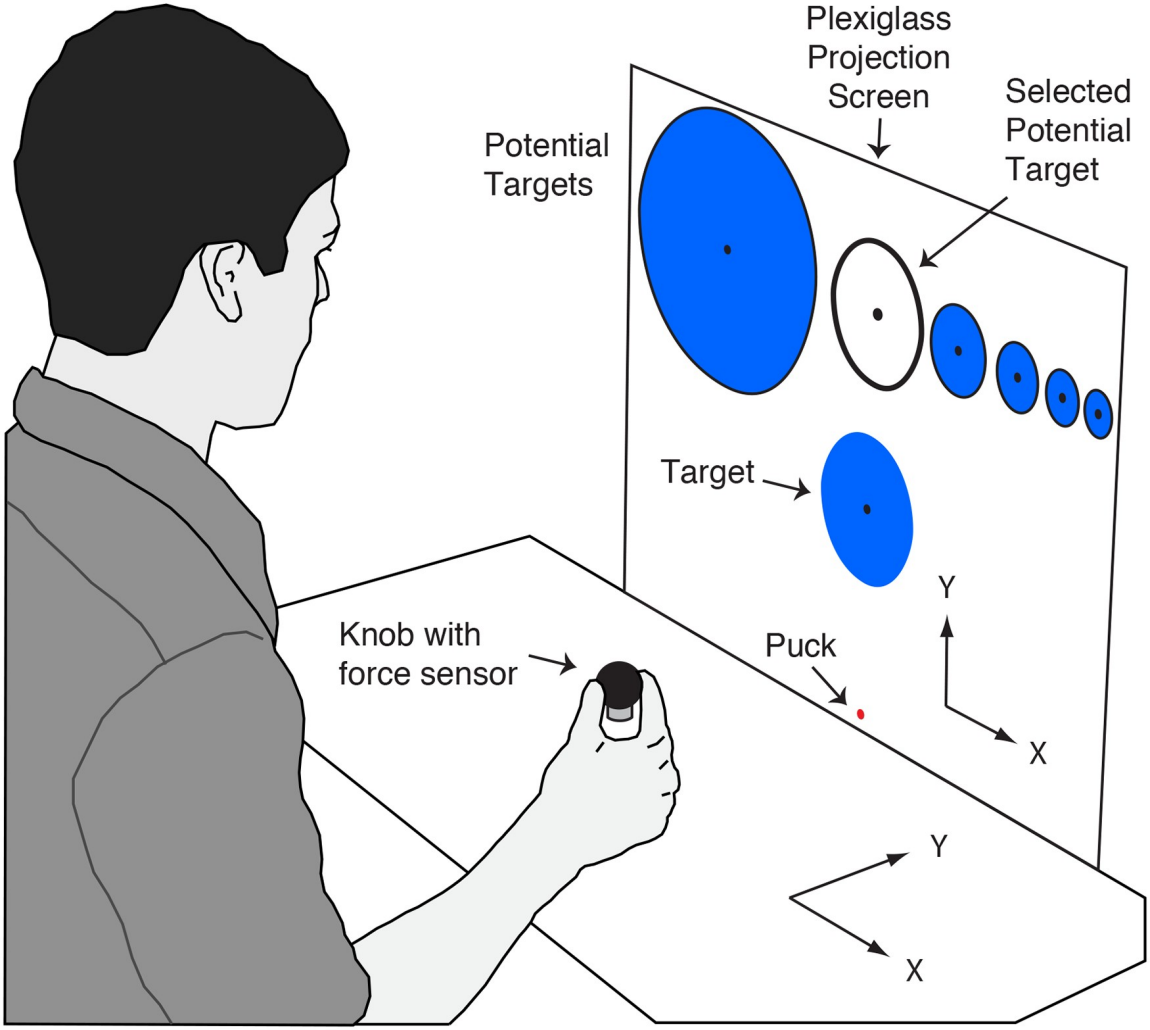
Exemplar display during target selection. The participant viewed potential targets at the top of screen and could select one of them using the left and right arrow keys on a keypad. The selected potential target was highlighted (filled white) and shown in the target position. The participant confirmed their target selection by pressing the down arrow key on the keypad. The radii of the 6 potential targets were 208, 104, 64, 48, 38 and 32 mm, and the corresponding rewards for a successful hit were 1, 2, 3, 4, 5 and 6 points. Note that in throwing trials, only the puck and the target were displayed.

In Experiment 1, participants performed a virtual throwing task in which they briefly applied a force to a rigid sensor to propel a virtual puck displayed on a vertical screen towards a circular target (see Methods and Fig 1). The puck acted as a damped point mass and over 200 trials participants tended to reduce their error as they learned the dynamics of the puck and improved their application of force. Indeed, we found that, for all participants, movement errors decreased approximately exponentially across trials. On each trial, the participant received a reward if the puck stopped within the target and the reward depended on the target size. There was six possible target sizes with the value of the targets increasing (integer values from 1 to 6) as the target size decreased. The experiment began with the largest and lowest value target and after every fifth trial, participants were given the opportunity to select from the range of targets smaller than the current target or stay with the current target (Fig 1). Critically, participants could not select a larger target and therefore selecting a smaller target involved a commitment. We included this constraint because we wanted to guard against participants ‘taking a chance’ on a smaller target in the knowledge that they could return to a larger target. Importantly, this constraint, by encouraging participants to delay switching to a smaller target, worked *against* our hypothesis that participants will select smaller targets based on expected *future* performance. In other words, if participants select targets that are tailored for future performance, as opposed to current or past performance, we can reasonably suggest that this selection is based on predicting future performance. We evaluated participants’ target choices using a model that could consider both past and future task variability. Although we fully expected that participants would select smaller and smaller targets as they performance improved, the key question relates to the timing of these selections and whether they are tailored to past or anticipated performance.

In Experiment 2, a second group of participants performed reaching movements under a visuomotor rotation (see Methods). After each trial, feedback was provided about whether the movement was a “hit” (i.e., the cursor controlled by the hand hit the target), a “miss”, or a “fault” (reaction time > 350 ms). After the first 30 trials, participants were told that they would be randomly assigned to one of three groups who would complete an additional 24, 150, or 216 trials. For each of these possible ‘exploitation horizons’, participants were asked to select a reward schedule with the value of a hit ranging from 60 to 100 points and the corresponding value of a miss ranging from 40 to 0 points. Participants were informed that they would receive a monetary payment proportional to the number of points they earned. Each participant was then told that they had, in fact, been assigned to a control group and, as a consequence, would perform 216 trials with the middle reward schedule of 80:20 (hit:miss). This allowed us to assay each participants’ full learning curve and then determine how many points they would have received for each selected reward schedule and time horizon. Based on the hypothesis that participants anticipate future improvements in performance, we predicted that as the length of exploitation horizon increased, participants would select reward schedules that increasingly favour hits over misses. We further predicted that, within each horizon, the selected reward schedule would correlate, across participants, with their actual performance.

## Results

### Experiment 1

#### Learning analysis

**Fig 2:**
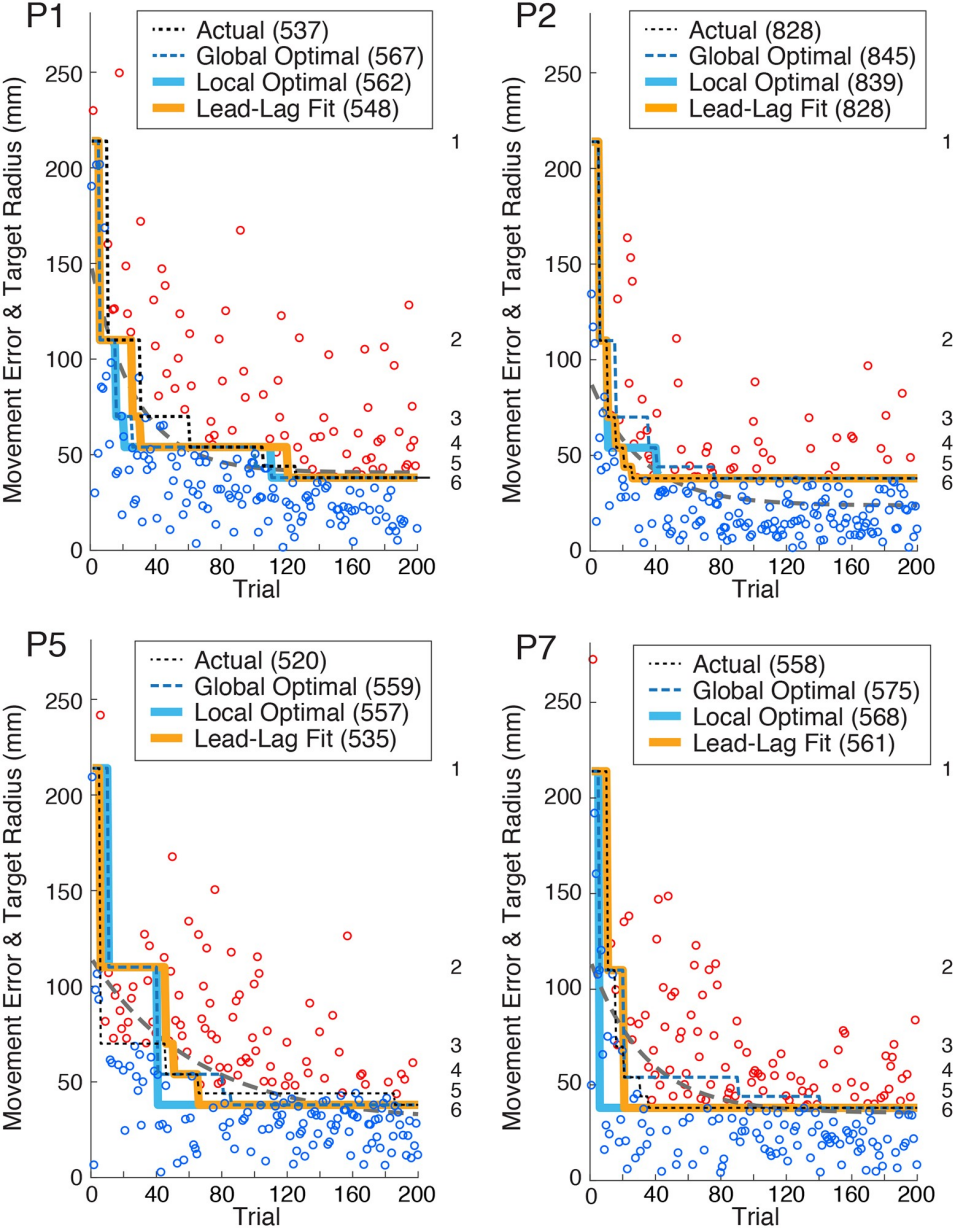
Movement errors (circles) and actual target selections (dotted black lines) as a function of trial for four representative participants. Target numbers are shown on the right of each panel. For each participant, target selections made by three models are shown: global optimal, local optimal, and lead-lag. Points earned by the participant and by the three models are shown in parentheses at the top in each panel. Movement error is color-coded by success (blue = hit, red = miss). Dashed gray line indicates fit of movement error to an exponential function of the form *ae*^*bx*^ *+ c*.

To quantify performance, we computed the movement error on each trial, defined as the resultant distance between the final position of the center of the puck and the center of the target. The circles in Fig 2 show movement error as a function of trial for four participants selected to illustrate the range in performance we observed. The dotted black line represents the radius of the target selected by the participant (except for the first block of 5 trials where the largest target was selected for them). The circles are colour-coded depending on whether the puck landed in the target (hit, blue) or not (miss, red). For all participants, movement error was initially large and then decreased across trials. The dashed gray lines in the figure show exponential fits to the error data. A paired t-test across participants revealed a significant difference (t7 = 6.0, p < .001) in movement error from the first block (M = 135 mm, SE = 17 mm) to the last block (M = 35 mm, SE = 4 mm), indicating that performance improved during the task. Note that half of the participants selected all 5 of the smaller targets during the experiment and the other half selected 4 of these targets, with three of these participants omitting the smallest target and one omitting the second largest target.

**Fig 3:**
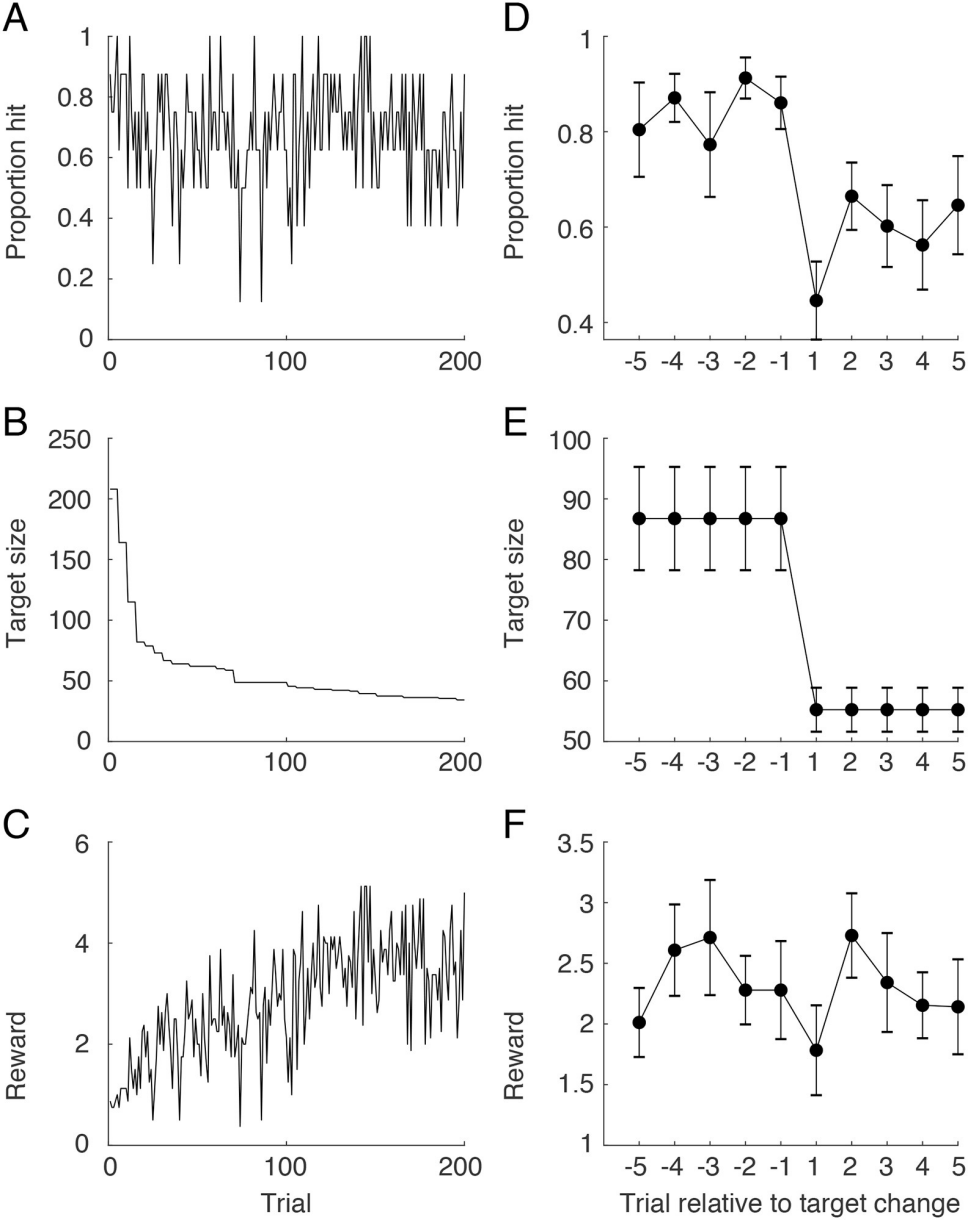
(A-C) Proportion of hits, selected target size, and reward achieved as a function of trial over the entire experiment, averaged across participants. (D-F) The same variables shown for the for the 5 trials before, and 5 trials after, a target switch, collapsing across all switches within a participants and then averaged across participants. Vertical bars represent ± 1 SE.

The left side of Fig 3 (panels A-C) shows the average proportion of hits, selected target size, and reward achieved on each trial over the entire experiment (200 trials). The right side (panels D-F) show these same variables for the 5 trials before, and 5 trials after, a target switch (collapsing across all switches). The results shown in the figure indicate that participants tend to keep hit rate approximately constant throughout the experiment but that there is a transitory dip in hit rate after a target switch. Note that there is a strong dip on the very first trial after a switch which is likely due to motor forgetting linked to the time delay involved in selecting the new target. In contrast, whereas the reward tends to increases over the entire experiment, no obvious increase in reward is observed immediately after a switch.

#### Optimality analysis

To assess how effectively participants selected targets, we first determined the ‘global optimal’ target selection scenario for each participant, which considers each participant’s performance over the entire experiment. This involved using a brute force approach that computed the number of points (i.e., expected reward) the participant would receive, based on their actual performance, for all possible target selection scenarios (>10^6^), subject to the same two constraints imposed in the experiment: (1) the target in the first block was the largest, and (2) at the start of a given trial block, a target larger than the currently selected target could not be selected. The dashed blue lines in Fig 2 show each representative participant’s global optimal target selection scenario. For half of the participants, the global optimal solution involved selecting all 5 of the smaller targets, and for the other half this solution involved selecting 4 of these targets.

Although both the participants and the global optimal model occasionally omitted a target (as noted above), most of the targets selected by the participants were also selected by the model. As illustrated in Fig 2, the trial block at which a participant first selected a given target could either lead or lag the trial block at which the global optimal model selected that same target. Participant P1 showed a clear pattern of lagging behind the global optimal model; i.e., they consistently selected specific targets after the global optimal model selected these targets. In contrast, participants P2 and P7 showed the opposite tendency, selecting specific targets before the global optimal model. A less consistent pattern was exhibited by participant P5, who initially selected smaller targets than the global optimal model, but later tended to select larger targets than the optimal model.

**Fig 4:**
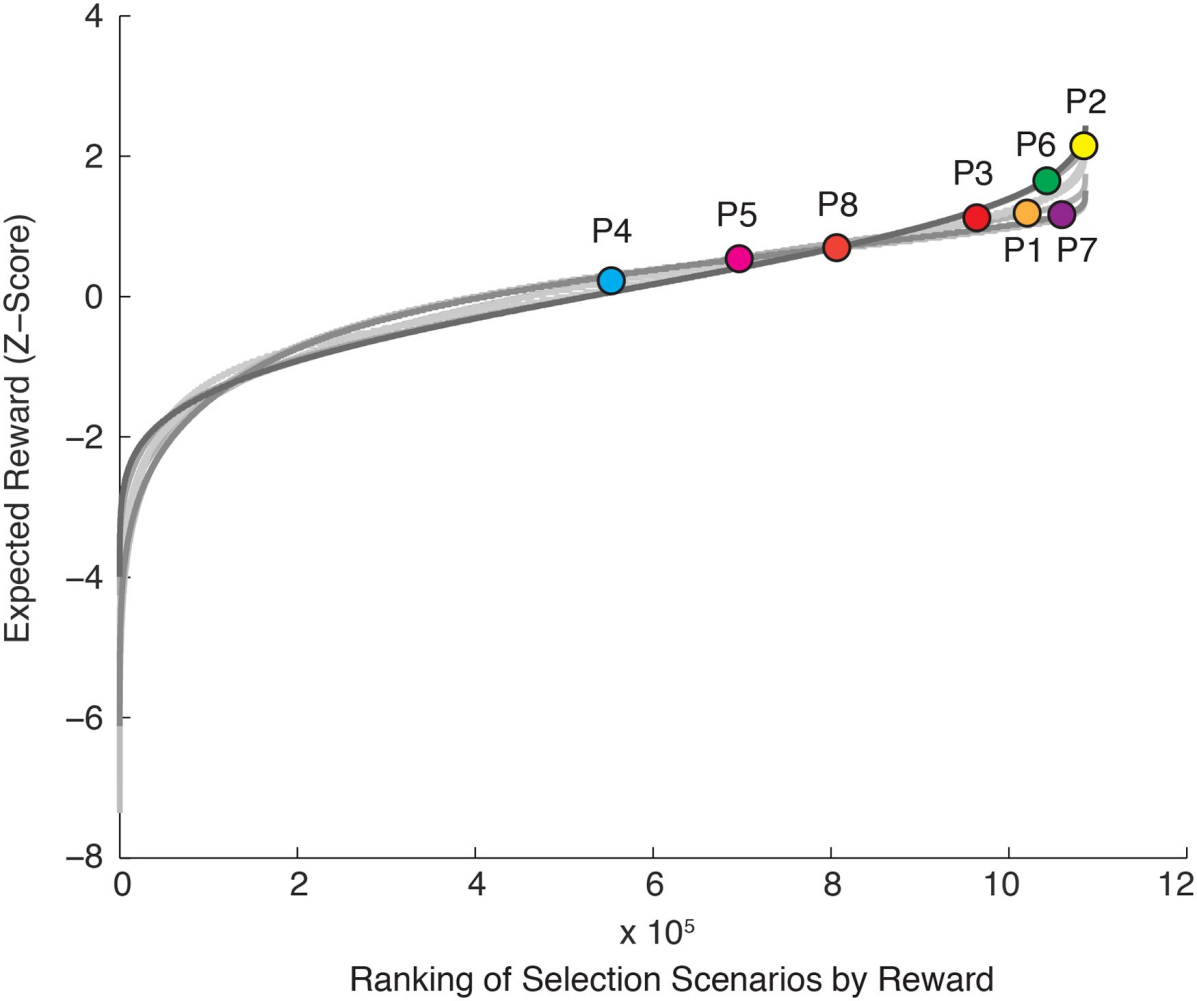
Curves show, for each participant, the expected reward—Represented as a z-score—For all possible target selection scenarios, as a function of expected reward ranking. The circles show the target selection scenario selected by each participant. Five participants exhibited a target selection scenario that was in the top 11 percent of all possible selection scenarios, and all participants exhibited a target selection scenario that was in the top half of possible selection scenarios.

The curves in Fig 4 show, for each participant, the expected reward as a function of all possible target selection scenarios, ranked in terms of expected reward. Note that we represented the expected reward as a z-score so that we could visually compare the curves from different participants. Five of the participants had target selections that were in the top 11 percent of the possible selection scenarios. The target selections of the other three participants were less optimal, but were all in the top half of possible selection scenarios. A paired-samples t-test comparing the optimal number of points each subject could have received (M = 618, SD = 175) against the actual number of points earned (M = 563, SD = 149) revealed that participants had a significantly lower (t_7_ = -2.65, p = .033) point total than optimal, with an average difference of 55 points (equivalent to $1.10). We also determined the percentile rank of the target decisions each participant made among all possible decision permutations. On average, participants scored in the 83^rd^ percentile of all decisions (SD = 18).

Whereas the global optimal model considers performance on all 200 trials and, critically, all decision points simultaneously, to find the best set of target selections, an arguably more biologically feasible model would be constrained to only consider past and future performance at each successive decision point (i.e., every 5 trials), independent of the other decision points. We refer to this model as the ‘local optimal’ model. The model was constrained such that the number of previous trials (lags) and the number of future trials (leads) were both constant across decision points, with the exception where these numbers were limited by the available number of previous or future trials, respectively. Therefore, at each decision point, the model calculates the reward that would be obtained over the trials spanned by the lag and lead, and chooses the target size that would maximize this reward, with the constraint that one can only switch to a smaller target or retain the currently selected one. To find the local optimum model that optimized reward, we iterated through all possible leads and lags across the experiment. The solid blue lines in Fig 2 show, for each representative participant, the predicted target choices for the local optimal model (fit separately to each participant). On average, the local optimal model considered 14 previous trials (SE = 4.8) and 18 future trials (SE = 6.5). A paired-samples t-test also revealed that the number of points predicted by the local optimal model (M = 615, SD = 175) was significantly greater (t_7_ = -2.41, p = .047) than the actual number of points earned by the participants, with an average difference of 51 points (equivalent to $1.02).

### Lead-lag participant fitting

To further assess the extent to which participants took into account past and future trials in their target choices, we determined the local lead-lag model that best predicted the target selections that participants actually made. Specifically, for each participant, we found the combination of leads and lags that minimized the sum of the squared differences (over all 39 choices) between the target number selected for the model and the target number selected by the participant. For comparison, we also determined the best models—in terms of predicting participant target choices—that only considered leads (lead model) or only considered lags (lag model). If participants were looking both ahead and behind when making target selections, we would predict that the combined lead-lag model should result in a smaller squared error (i.e. reflect participant choices more accurately) when compared to the fits produced by either the lead model or the lag model. We used F tests to compare the lead-lag model with the lead model and the lag model, so as to take into account differences in the number of parameters used in the models.

We first compared the models when combining all participants while allowing separate lead and/or lag parameters for each participant. Thus, there was a total of 8 parameters for the lead model and the lag model (1 model parameter x 8 participants), and 16 parameters for the combined lead-lag model (2 model parameters x 8 participants). The combined lead-lag model provided a significantly better fit to the participants’ data than either the lead model (F_(8,296)_ = 18.84, p < 0.001) or the lag model (F_(8,296)_ = 69.46, p < 0.001). When the models were compared for each participant separately, we found that for 7 out of 8 participants the combined lead-lag model performed significantly better than the lead model alone (at a p < 0.05 level), and that for 6 of 8 participants the lead-lag model performed significantly better than the lag model alone (at a p < 0.05 level). The orange lines in Fig 2 show, for each representative participant, the target selections produced by the lead-lag model. When comparing the 8 parameter lead and lag models, we found that the sum of squared residuals was much smaller for the lead model (524) than the lag model (999), indicating that the lead model provided a substantially better fit than the lag model. By comparison, the sum of squared residuals for the 16 parameter lead-lag model (347) was considerably smaller. The mean absolute deviation—averaged across the 8 participants and 39 selection points—between the targets selected by the participants and the targets selected by the three models were: lag: 1.27, lead: 0.891, and lead-lag: 0.663. (Note that a value of 1 indicates that, on average, the model is off by one target; e.g., selecting target 6 instead of target 5).

**Fig 5:**
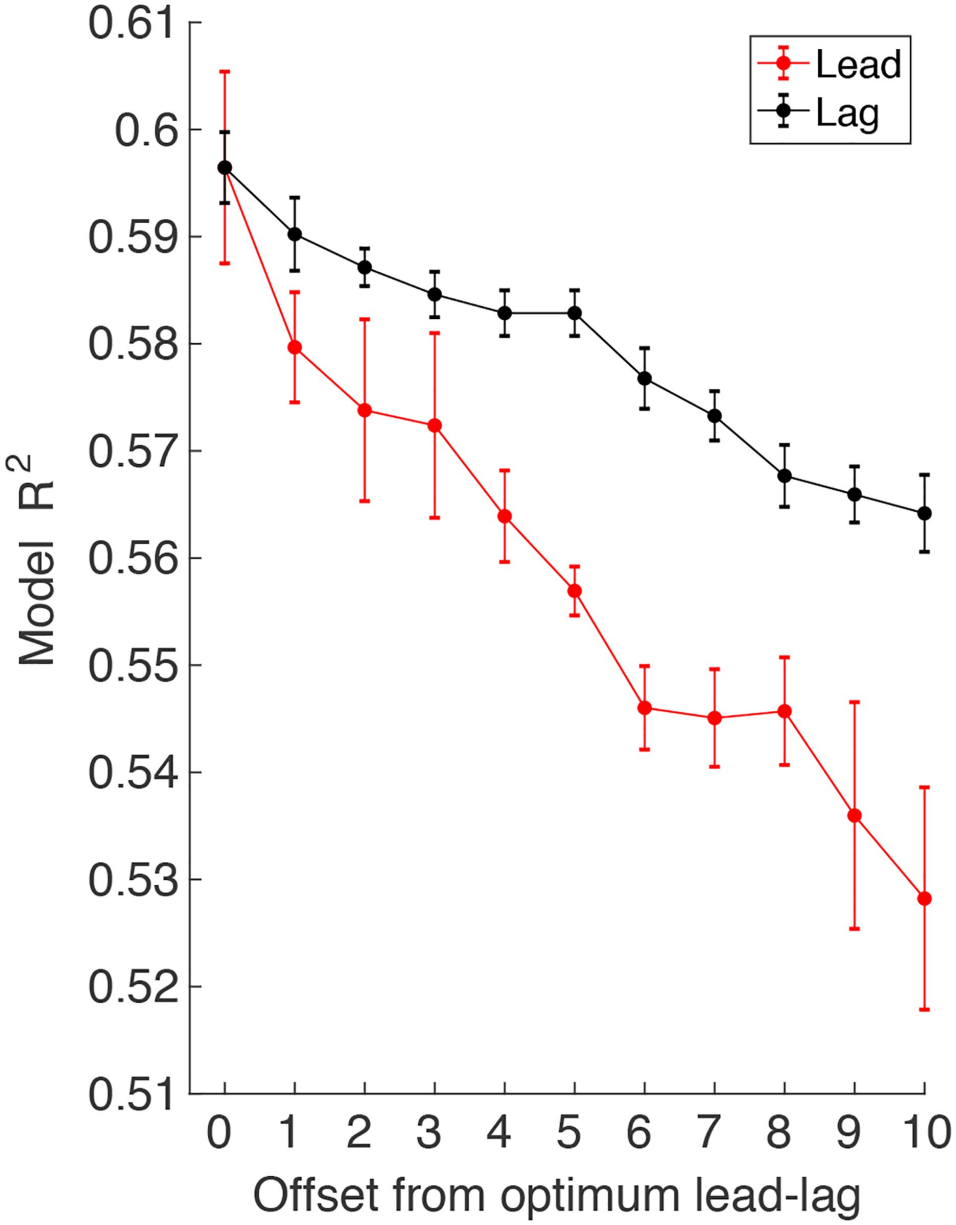
Sensitivity analysis of the lead-lag model fits. The variance explained (averaged across participants) is shown as the lead and lag are changed from the optimal lead-lag model for each participant. We averaged R^2^ across for positive and negative offsets up to 10 leads/lags. (Note that for participants with leads or lags less than 10, we took only the offsets we could calculate). The standard error bars were calculated by first removing the mean variance explained for each participant averaged over all leads and all lags (separately), so that the error bars reflect the variability of the decrease in variance explained with offset rather than the variability of the average variance explained across participants.

The lead-lag model indicated that, on average, participants looked ahead 38 trials (SE = 16.6) and behind 46 trials (SE = 13.2) when selecting targets. Fig 5 shows a sensitivity analysis of this lead-lag model fit as the lead and lag are offset from the optimal values. This figure shows that the fit is more sensitive to changes in the lead than in the lag. A paired sample t-test revealed that the points earned by the lead-lag model (M = 600, SE = 64) was not significantly different (t_7_ = -1.6; p = 0.15) than the actual points earned by the participants (M = 563, SE = 53), indicating that the model provided a good fit to the data. Taken together, these results suggest that participants took into account both past errors and predicted future errors when making target selection decisions during learning.

Our model assumes that there is no relationship between target selection and motor performance. That is, we assume that participants always try to minimize error to the same extent regardless of the size of the target currently selected. To assess this assumption, we compared movement errors, as a function of trial block, in our main experiment—in which participants selected increasingly smaller targets—with errors in our exploratory experiment in which the target size was always very small. (Note that for this analysis, we used the first 200 trials from the exploratory experiment.) If movement errors vary as a function of target size then we would expect to see a significant interaction between experiment and trial block. A two-way experiment (main versus exploratory) by trial block (40 blocks) mixed factor ANOVA failed to reveal a main effect of group (F_1,15_ = .79, p = .388) or an interaction (F_39,585_ = .759, p = .856) between block and group, which suggests that motor performance (i.e., error) was not affected by target size.

**Fig 6:**
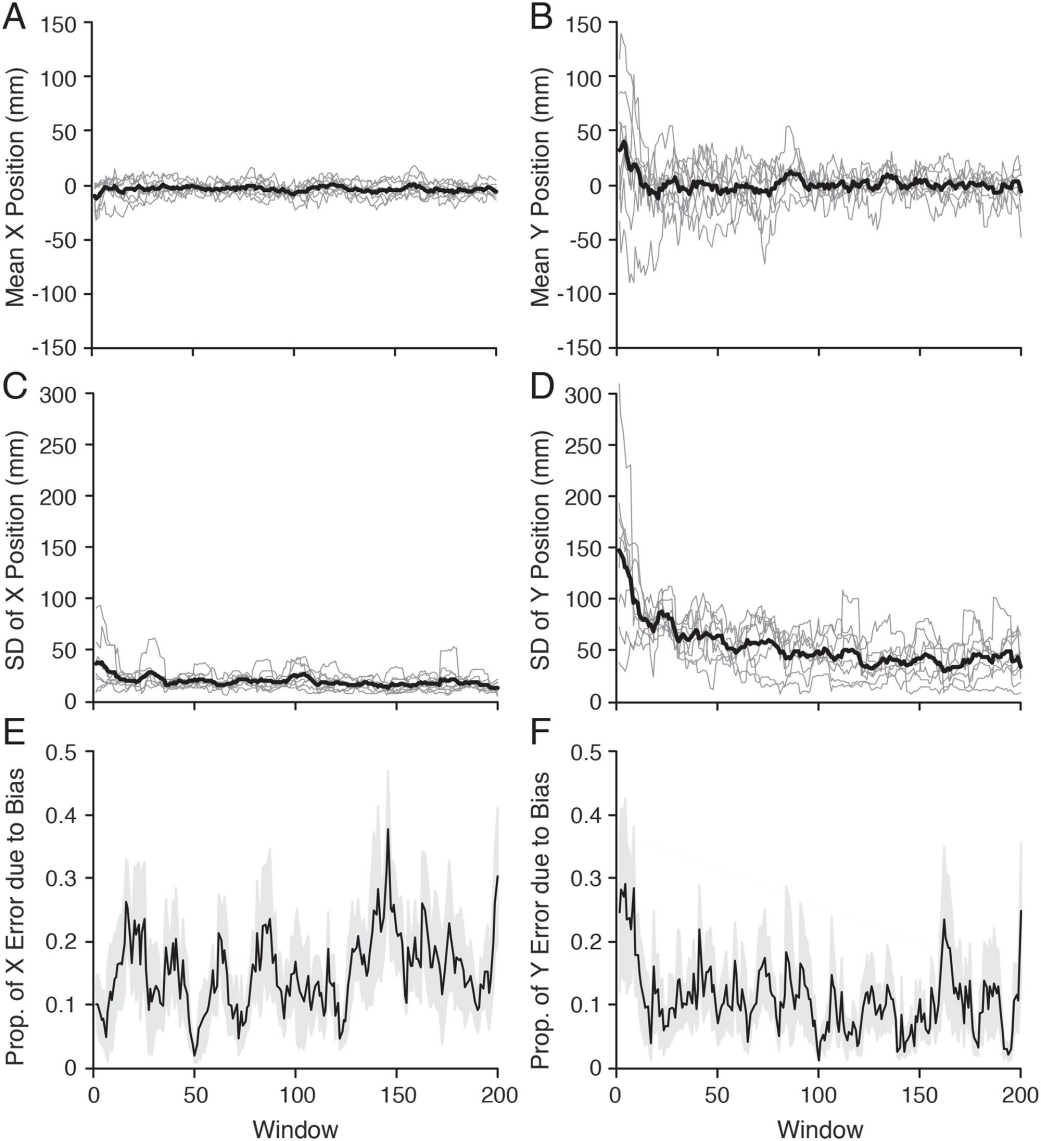
Mean x (A) and y (B) final puck positions and the corresponding x (C) and y (D) standard deviation (SDs) about these means, computed over a moving window of 9 trials. Thin gray lines represent individual participants and the thick black line represents the average across participants. The mean position provides an estimate of the bias and the SDs provide an estimate of the variance. Panels E and F show, for x and y respectively, the proportion of the mean squared error (MSE) accounted for by the bias, where MSE = (mean bias)^2^ + variance. The thick black line represents the average across participants and the height of the gray regions represents ±1 standard error.

We assessed performance in our task in terms of movement error, defined as the distance between the final position of the puck and the center of the target. This is appropriate since success or failure in the task is determined by this error. Performance can also be assessed by examining the bias and variance. (For a scalar error, the mean squared error is the (mean bias)^2^ + variance). In previous work examining well learned reaching tasks, the bias is close to zero [12,14,15]. However, in our learning task, we might expect that error reduction involves reducing both the bias and the variance. To assess how these different components of error changed in our task, we computed the final mean x and y puck positions and the corresponding standard deviation (SDs) about these means, in a moving window of 9 trials (Fig 6A–6D). As might be expected based on our task, the bias and variance were substantially larger in y than x. As shown in panel B, on average participants tended to overshoot the target during initial learning (thick black line), although there was considerable variability across participants in terms of this bias (thin gray lines). As can be visually appreciated, this bias was largely reduced to zero after about 20 trials. As shown in panel D, the SD in y gradually decreased across the first 100 trials. Fig 6E and 6F show, for x and y respectively, the proportion of the mean squared error (MSE) accounted for by the bias. Overall, the bias accounted for about 15–20 percent of the x MSE and closer to 10 percent of the y MSE. For the x position, this percentage did not change with learning. However, for the y position, the percentage was relatively high during the initial 20 trials before leveling off. Note that we also examined the correlation between x and y error over the same window (not shown). The correlation, averaged across participants, varied between -0.42 and 0.16 over the course of the experiment, and thus we did not observe any strong dependence between these errors.

Taken together, the findings from Experiment 1 indicate that participants take into account both their past and, critically, future performance when deciding between targets associated with different rewards. This suggests that people predict their future motor performance when making skill-based decisions.

### Experiment 2

**Fig 7:**
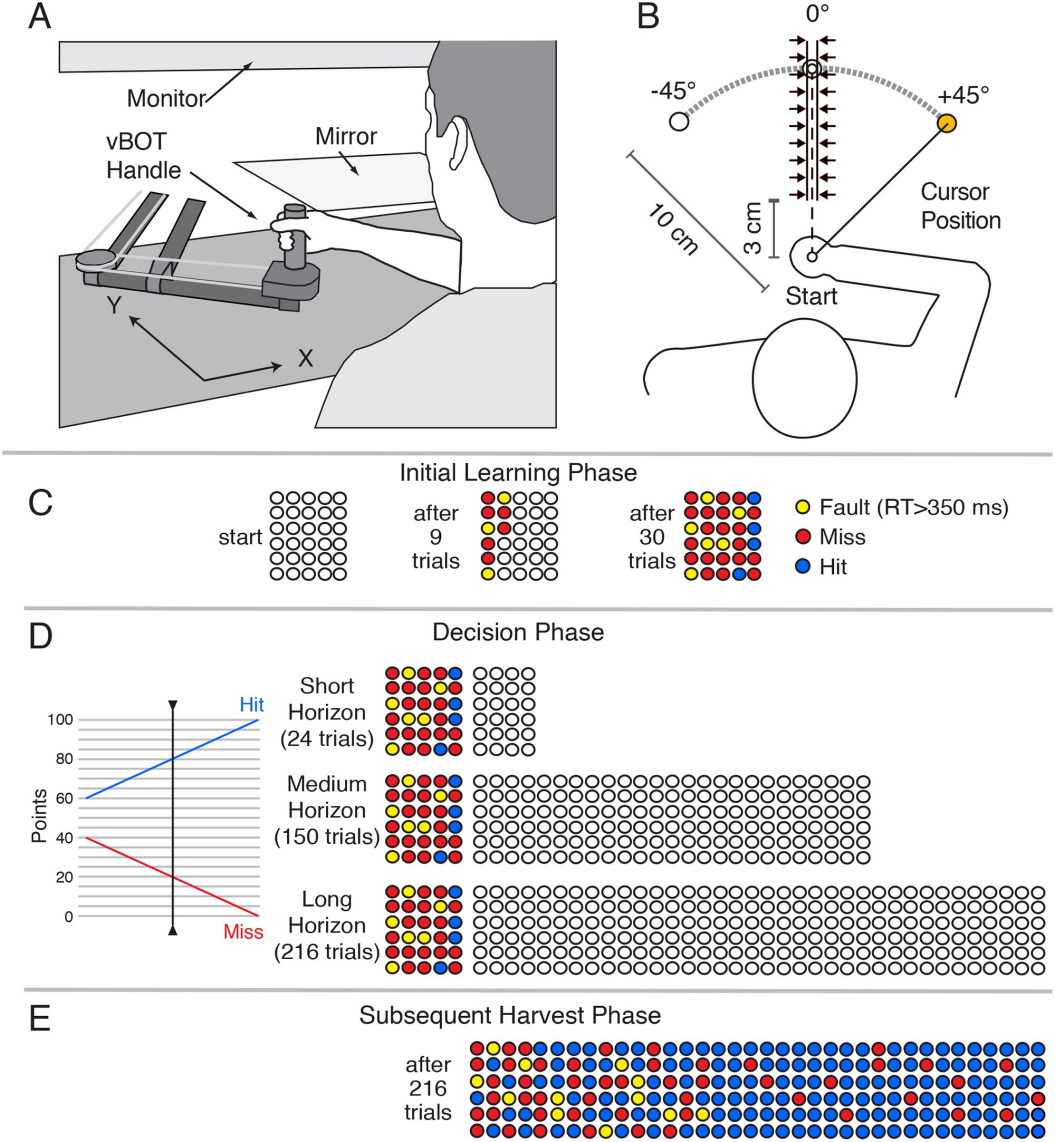
(A) Schematic of robotic device and virtual reality setup used in Experiment 2. (B) Top view showing the locations of the start location and three targets. A force channel, represented by the arrows, constrained the movement to a straight line path in the direction of initial movement. During visuomotor rotation trials, the position of the viewed cursor, controlled by the handle, was rotated 45° about the start location. (C) Circles representing the number of trials to be performed during the initial learning were displayed to the participants throughout this phase. These filled in as trials were completed, with the colour indicating a hit, miss, or fault (reaction time > 350 ms). (D) During the decision phase, participants viewed filled circles showing their initial learning performance and empty circles showing the number of trials for three different horizons. Using the ‘slider’ shown at the left, they selected reward schedules for each horizon. (E) Performance associated with a representative participant’s subsequent (Long Horizon) harvest phase.

In Experiment 2, we examined participants’ ability to predict future performance using a visuomotor adaptation task in which participants generated target directed out-and-back reaching movements in the horizontal plane. In each trial, the participant moved a cursor—controlled by a handle attached to a robotic device [23]—from a start position out to one of three randomly selected targets and back again in a continuous movement (Fig 7A). When the visuomotor rotation was implemented, the viewed position of the cursor was rotated 45 degrees about the start position. To limit the use of cognitive strategies to adapt to the rotation, the reach had to be initiated within a reaction time (RT) of 350 ms after target presentation [24,25]. In addition, to prevent online corrections in movement direction, once the hand was displaced 3 cm from the start position, a ‘force channel’ was implemented in line with the current movement direction (Fig 7B). After each trial, feedback was provided about whether the movement was a “hit” (i.e., the cursor hit the target), a “miss”, or a “fault” (RT > 350 ms).

Participants first completed a series of baseline trials, without a visuomotor rotation, to familiarize them with the task. Participants then completed an initial 30 trials with the visuomotor rotation (Fig 7C). They were then told that they would be randomly assigned to one of three possible groups and, depending on the group, complete either an additional 24, 150, or 216 trials. For each of these ‘horizons’, the participant was asked to select a reward schedule with the value of a hit ranging from 60 to 100 points and the corresponding value of a miss ranging from 40 to 0 points (Fig 7D). Thus, participants could never lose points by missing. (An RT fault always resulted in 0 points.) Participants were informed that they would receive a monetary payment proportional to the number of points they earned. Note that a reward schedule of 60:40 (hit:miss) would be more profitable if performance over the horizon was poor, whereas a reward schedule of 100:0 would be more profitable if performance over the horizon was excellent. Once the three rewards schedules were selected, the participant was then told that they had, in fact, been assigned to a control group who would perform 216 trials with the middle reward schedule of 80:20 (hit:miss). They then performed these additional trials (Fig 7E).

**Fig 8:**
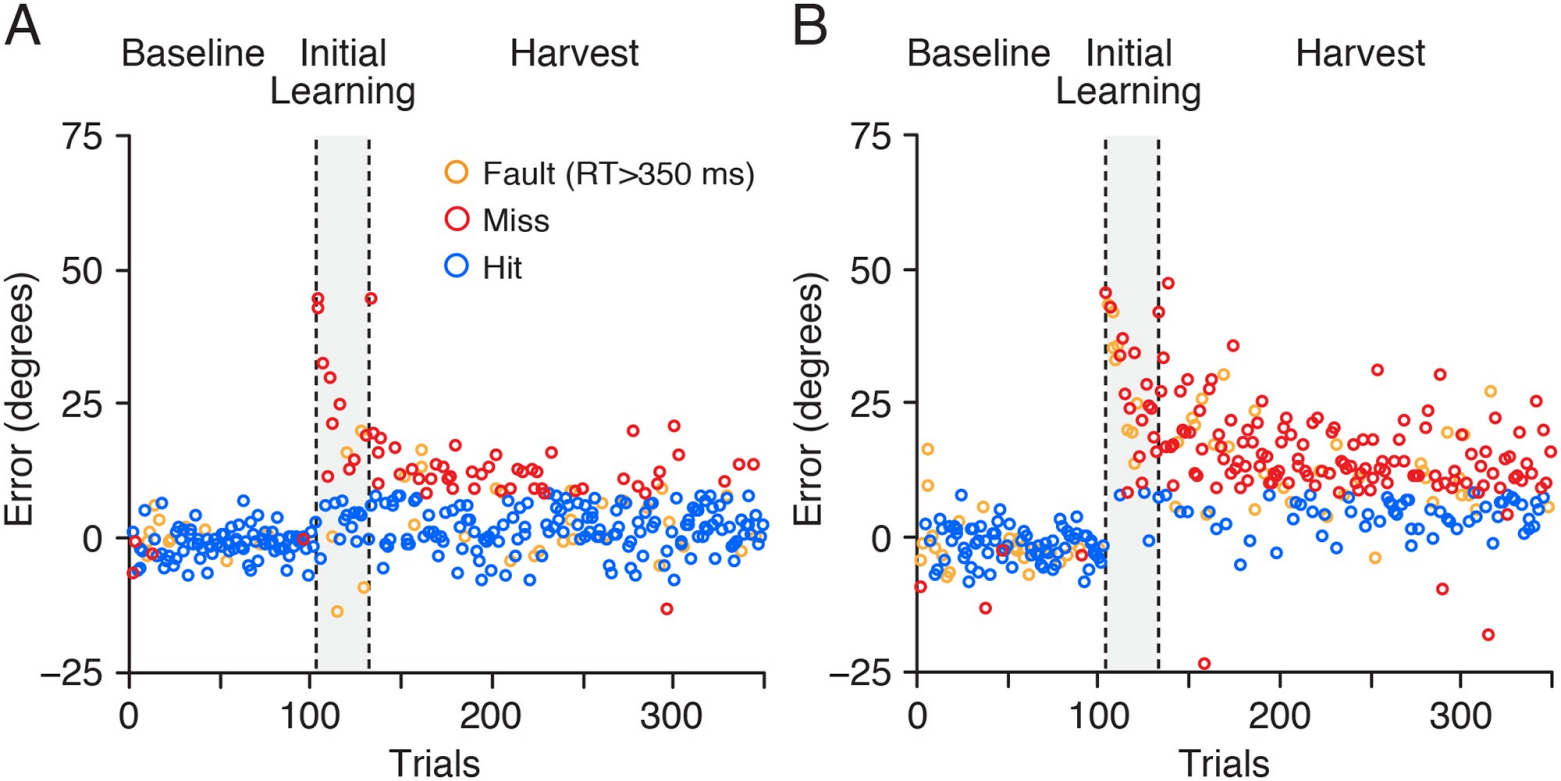
Angular reach error as a function of trial during the baseline, initial learning (gray region), and subsequent harvest phases shown for two participants: A good learner (A) and a poor learner (B). Circles are colour coded depending on whether the trials resulted in a hit, miss, or fault (RT > 350 ms). Note that occasional misses occurred due to undershooting the target even though the directional error was close to zero.

**Fig 9:**
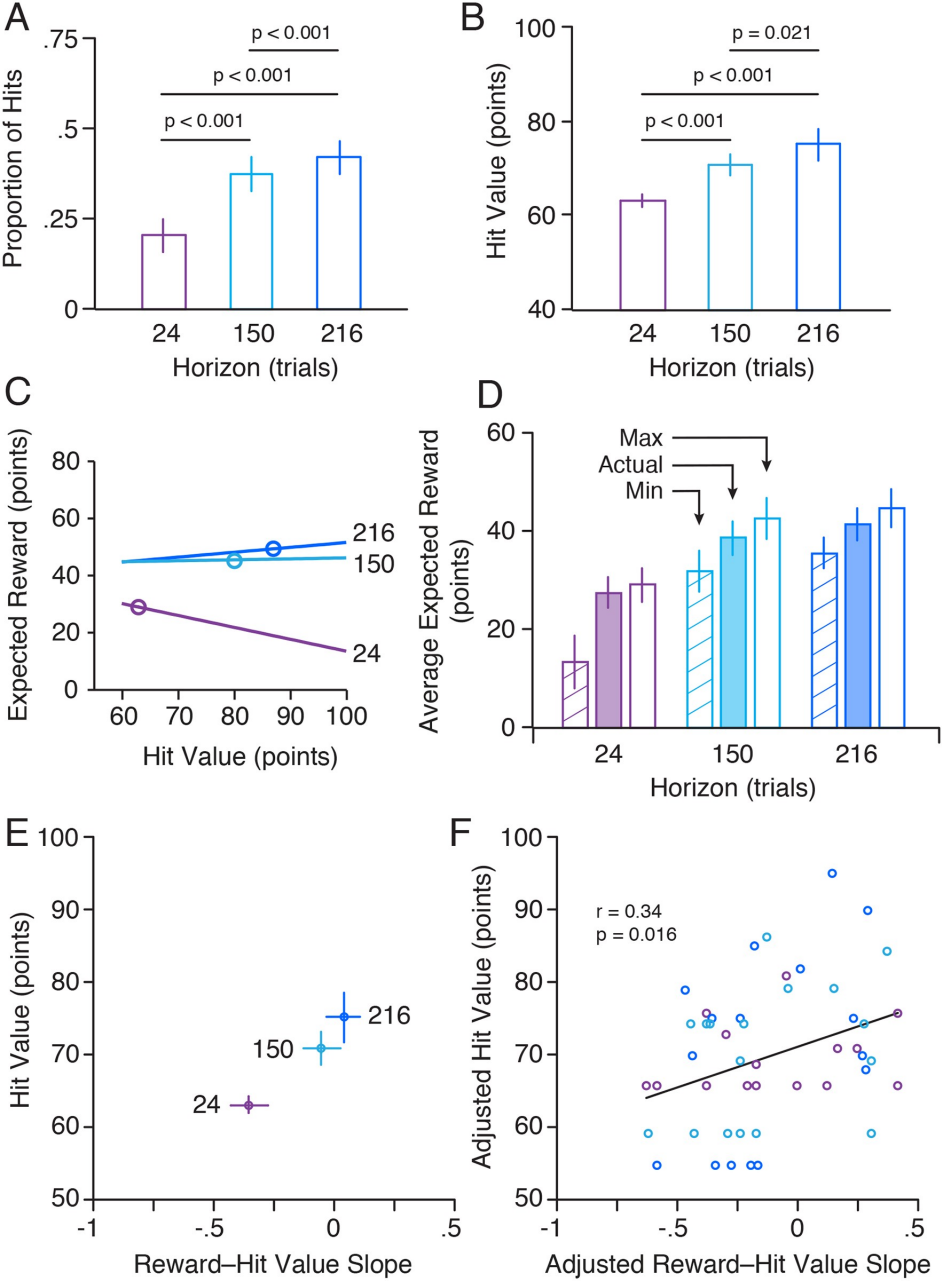
(A) Mean proportion of hits, averaged across participants, for the short, intermediate, and long horizons (i.e., over the first 24, 150, and 216 trials of the harvesting phase). (B) Mean selected hit value, averaged across participants, for the three horizons. (C) The three lines show, for a single participant, the relationship between hit value and expected reward for each horizon (see numbers). The corresponding circles show the actual hit values selected by the participant for each horizon. (D) Shaded bars show the average reward, earned by participants, for each of the horizons. The open and striped bars show the corresponding maximum and minimum rewards that could have been achieved depending on hit value selection. (E) Average selected hit values as a function of the average slope of the relationship between expected reward and hit value (i.e. slopes of lines in C) for each horizon. (F) Relationship between hit value and the reward-hit value slope, after removing average effects of the horizon (shown in E). Each circle represents a single participant and horizon, and is colour coded by horizon (see E).

Fig 8 shows angular reach error as a function of trial for two representative participants. Although both participants exhibited an approximately exponential decrease in error over the initial learning and harvest phases, the participant shown in panel A exhibited better learning and more frequently hit the target (blue circles) during the harvest phase. Fig 9A shows the mean proportion of hits, averaged across participants, for the short, intermediate and long horizons (i.e., over the first 24, 150, and 216 trials of the harvesting phase). As expected, the proportion of hits increased as the length of the horizon increased and performance improved. A repeated measures ANOVA revealed a significant effect of horizon on the proportion of hits (F_2, 32_ = 34.0; p < 0.001). Follow up paired t-tests revealed significant differences between all three pairwise comparisons (24–150: t_16_ = -5.23; p < 0.001, 24–216: t_16_ = -6.28; p < 0.001, 150–216: t_16_ = -6.93; p < 0.001), which remain significant when corrected with the Holm-Bonferroni method.

Fig 9B shows the average hit value, selected by the participants for the three horizons. A repeated measures ANOVA revealed a significant effect of horizon on hit value (F_2, 32_ = 15.8; p < 0.001) and paired t-tests revealed significant differences between all three pairwise comparisons (24–150: t_16_ = 4.035; p < 0.001, 24–216: t_16_ = 4.333; p < 0.001, 150–216: t_16_ = 2.562; p = 0.021), which remain significant when corrected with the Holm-Bonferroni method. These results indicate that participants anticipated the fact that their performance would improve across the harvesting phase when selecting the reward structure.

To determine the optimal hit value, we calculated, for each participant and horizon, the reward the participant would have obtained—based on their actual performance—as a function of hit value. This procedure is illustrated, for one participant, in Fig 9C. Note that the expected reward is a linear function of the hit value. Consequently, the optimal hit value was always either 60 or 100 depending on whether the slope of the relationship between hit value and expected reward is negative or positive. For this participant, the optimal hit value (i.e., the hit value yielding the largest reward) was 60 for the short horizon and 100 for the intermediate and long horizons. The circles represent the hit values selected by this participant for each horizon. For the short horizon, the participant selected a hit value of 63, which was close to optimal. For the intermediate horizon, the participant selected a hit value of 80. Although this was far from optimal (100), because the slope was only slightly greater than 0 (0.03) the consequence in terms of reward was small. For the long horizon, the participant selected a hit value of 87, which was closer to the optimal hit value.

The shaded bars in Fig 9D show the reward, averaged across participants, for each of the horizons, and the open and striped bars show the corresponding maximum and minimum rewards, respectively, that could have been achieved depending on hit value selection. Paired t-tests revealed that the maximum (or optimal) reward was greater than the actual reward for all three horizons (Short: t_16_ = 2.71; p = 0.015, intermediate: t_16_ = 3.66; p = 0.002, long: t_16_ = 3.15; p = 0.006), differences that remained significant when corrected with the Holm-Bonferroni method. However, as can be visually appreciated, participants made close to optimal selections for the short horizon, whereas the difference between the actual reward and the maximum reward was greater for the two longer horizons (although in all cases, the actual reward was closer to the maximum reward than the minimum rewards).

Fig 9E shows how, on average, the selected hit values varied with the slope of the relationship between expected reward and hit value (i.e. slopes of lines in Fig 9C) across the three horizons. As illustrated in the figure, both the average slope and the average selected hit value increased with horizon length. Thus, there was a clear correlation, across horizons, between the selected hit value and the slope. Note that the slope, for a particular participant and horizon, provides a index of that participant’s performance across that horizon. A positive slope—where the optimal hit rate is 100—is indicative of good performance, whereas a negative slope—where the optimal hit rate is 60—is indicative of poor performance. If participants’ choices about reward were linked to their performance, then—for each horizon—we would expect to see a positive correlation between selected hit value and the reward–hit value slope. To assess this relationship, we combined the results from the three horizons after removing the average effects of the horizon (shown in Fig 9E). Specifically, for both the hit values and slopes, we subtracted the mean from that horizon, and then added back the grand mean to obtain adjusted hit values and slopes. As shown in Fig 9F, a positive correlation (r = 0.34; p = 0.016) between the adjusted slope and the adjusted hit value was observed. To assess the robustness of this correlation, we also calculated the correlation with 10-fold cross validation. To do this we performed linear regression with Monte Carlo sampling 1000 times to calculate the predictive SSE. We also did this for a reduced model (fit by just the mean). From this, we calculated the predictive *r* and associated p-value. This stringent test gave a correlation coefficient (r = 0.28) that remained significant (p = 0.0458). Thus, both across horizons and across participants within horizons, we see that decisions about rewards are sensibly linked to future, actual performance.

Note that participants also selected hit values for the baseline trials. Specifically, at the start of the experiment, participants first performed 30 trials without a visuomotor rotation and then used the slider to select a reward schedule for an additional 102 baseline trials, while viewing filled circles depicting their performance over the first 30 trials and unfilled circles representing the additional trials (as in Fig 7D). During these additional baseline trials, they received points according to their selections. The mean hit rate for these baseline trials was 0.90 (SE = 0.03) and the mean hit value selected by participants was 89.8 (SE = 2.4), which is well above the values selected for the three horizons of visuomotor rotation trials (Fig 9B). These results are consistent with our finding, from the visuomotor rotation trials, that decisions about rewards are sensibly linked to performance. The average reward that participants received during these baseline trials was 81.7 (SE = 3.3). The average maximum reward participants could have received—which for all but one participant would have been earned by selecting a hit value of 100—was 90.1 (SE = 2.9), and the average minimum reward they could have received was 57.8 (SE = 0.8). This result is also consistent with our finding, from the visuomotor rotation trials, that participants selected conservative hit values that yield a little less than optimal reward, but in the optimal direction.

## Discussion

The aim of the current study was to test whether, when learning a novel motor task in which movement errors tend to decrease across trials, participants can take into account future performance (i.e., reduced errors) when making decisions that will determine future performance-related reward. We addressed this question in two experiments involving two different motor learning tasks. In the throwing task employed in Experiment 1, participants were given the opportunity to make decisions about reward—by selecting target size which was inversely related to the reward obtained for a successful hit—throughout learning. In the visuomotor adaptation task used in Experiment 2, participants first performed a set of initial learning trials and then made decisions about reward for different possible horizons; i.e., additional trials they might have to subsequently perform. We predicted that, in both of these tasks, participants would not base their choices solely on current and/or recent task performance but would also consider their predicted future performance.

In Experiment 1, we found that all participants improved on the task in that their reach errors, relative to the centers of the targets, decreased across trials. This reduction in movement error was complemented by participants making use of (i.e., selecting) either all or all but one of the smaller targets. Overall, participants’ decision making in terms of target selection was quite effective in the task. On average, participants obtained ~90 percent of the total points achieved by the global optimal model. Moreover, participants’ target selection scenarios were, on average, in the upper 20 percent of all possible scenarios. This suggests that participants readily appreciated the nature of the task and how target choice could affect reward. This interpretation is consistent with previous work indicating that people readily appreciate the mapping between target size and penalties and rewards in the context of reaching [12,14–16].

To assess the extent to which participants considered past and future performance when selecting targets, we tested models in which the target selection at each decision point was based on movement errors from the past (lag model), the future (lead model), or a combination of the past and future (lead-lag model). For each model, we found, for each participant, the lag, lead, or combination of lead and lag that most closely matched the participant’s target choices. Overall, the lead model outperformed the lag model, but both of these models were significantly poorer than the lead-lag model. This suggests that participants’ choices took into account future performance, in addition to past performance, when selecting targets. While it is unsurprising that participants selected increasingly smaller targets throughout the experiment, it is not obvious that the decision to reduce target size would be based not only on current performance but also on expected future performance. This is despite the fact that our task, by not allowing participants to revert to a larger target, likely promoted conservative choices that would, if anything, delay switching to a smaller target.

All of the models that we evaluated used participants’ actual past and future errors, and thus assume that participants had complete knowledge of these errors. Although it seems unlikely that participants remembered all their past errors, it seems entirely plausible that they could maintain some summary statistics of these errors, such as a running average and a running variance. Recent work suggests that the brain, in addition to storing a memory of past actions [26], may also separately store a memory of past errors [27] or anticipate future errors [28]. Whereas participants observed past errors, future errors had to be estimated. There are a number of approaches that could be implemented to predict future errors; for example estimating a noisy exponential based on past performance and extrapolating. Indeed, any model of learning [e.g., 29,30,31], including models that represent the statistics (e.g. prior) of past learning experiences, could be adapted for this purpose. Importantly, implementing such models, assuming they provide a good fit, would not alter our basic finding that participants consider both past and future performance when selecting targets. Previous work has shown that when estimating variance, or uncertainty, participants weight recent trials more heavily [17,19]. In our models, we chose, for simplicity, to equally weight all errors in a given time window. However, the models nevertheless capture recency effects by testing different lag windows.

The lead-lag model indicated that participants looked ahead, on average, 38 trials. In theory, if participants’ errors decreased monotonically, the optimal solution would be to only consider 5 trials into the future at each decision point (i.e., the movements until the subsequent decision point). The fact that our participants considered a longer horizon presumably reflects the fact that their learning curves were not monotonic but, instead, were quite noisy.

Our model assumes that there is no relationship between target selection and motor performance. That is, we assume that participants always try to minimize error to the same extent regardless of the size of the target currently selected. Although there is evidence that practicing a task at an optimal difficulty improves performance [32], our results suggest that movement error was not influenced by participants’ target choices. Specifically, we did not observe any differences in movement errors across trials between participants in the main experiment (who selected targets of varying size) and participants in our exploratory experiment who were presented with a very small target throughout. This result is perhaps not surprising given that optimal performance—in terms of reward—in our task involved selecting targets that were always challenging, resulting in frequent misses. Therefore, it is likely that participants were highly motivated to minimize error.

The results of Experiment 2 provide additional support for the hypothesis that participants factor into account future, improved performance when making decisions about reward. We found that participants selected more challenging reward schedules (i.e., greater hit values) as the length of the horizon increased, indicating that they correctly predicted that their performance would improve across trials. We also found that, for the shortest horizon, participants selected close to optimal reward schedules. For the longer horizons, perhaps not surprisingly, participants’ choices were less optimal but still in the optimal direction. We also examined the relationship between hit value selection and the sensitivity of reward to this choice—characterized by the slope of the relationship between hit value and expected reward—which depends on performance. We found that when the slope was negative, indicating poor performance, participants tended to appropriately select lesser hit values. In contrast, when the slope was positive, indicating good performance, participants tended to select greater hit values.

One possible interpretation of the results of Experiment 1, is that participants, rather than basing their target selections on predicted improved performance, base their selections on current performance but overestimate this performance, which would lead them to select smaller and smaller targets. This interpretation requires that participants consistently overestimate their current performance level throughout learning. One argument against this alternative interpretation is that previous studies indicate that people, when performing a familiar reaching task, do not overestimate their performance, which in this task is determined by movement variability [12,14,15]. Even when variability is artificially changed, people quickly adjust their estimate so as to maintain an accurate evaluation of their performance [17]. The results of Experiment 2 also argue against this interpretation. We found that, when selecting reward schedules for different horizons after initial learning, participants successfully anticipated that their performance would get better and better across trials. Had the participants based their selections of reward structures on an overestimate of their current performance—rather than predicted future performance—they would presumably have selected the same reward schedule for all horizons.

Numerous studies have investigated the evaluation of immediate and future rewards in the context of reward-guided learning and decision-making, often with a focus on the underlying neural processes involved [33,34]. This work has shown that participants can learn about the properties of changing environments and reward contingencies, and use this information to intelligently guide reward-oriented behaviour. For example, it has been shown that humans can assess the changing volatility of the reward environment so as to make optimal decisions [35]. Similarly, studies of patch foraging have shown that participants can represent the value of the current patch and potential new patches in order to make optimal decisions regarding the exploration-exploitation trade-off [36–39]. Whereas this previous work has shown that participants can learn about changes in the reward environment, the current study demonstrates that participants can anticipate changes in their own performance when making performance-based decisions about reward.

Previous work has shown that when making movement-related decisions (e.g., where to aim), people can take into account errors in their steady-state performance so as to optimize rewards [12]. The main advance of the current work is the demonstration that people can take into account predicted *changes* in performance, associated with motor learning, to increase reward. Note that in well learned motor tasks, such as simple reaching, the bias (e.g., mean reach direction) is minimal and errors are well captured by the variance about the mean. In contrast, errors during motor learning typically arise from both bias and variance. Our results indicating that participants anticipate decreasing errors during motor learning, suggests that they consider both the bias and the variance. Whereas our work examined relatively simple motor learning tasks, the ability to predict future performance would clearly be advantageous in many learning contexts. Moreover, this ability could provide a basis for making decisions about when to invest in learning, ranging from a programming language to a new tennis grip. Across many domains, most of us will quite often try our hand at learning a new task or new content, and along the way may consider whether it is worthwhile to continue based on an evaluation of how well we will learn, how long it will take, the effort (and any other costs) that will be involved, and the rewards we might obtain. In principle, understanding the bases of such decisions could be informative in terms of designing learning situations that allow people to make optimal investment decisions.

## Methods

### Experiment 1: Throwing task

#### Participants

Eight participants (3 female) between the ages of 18 and 26 years old (M = 21.4) were recruited to take part in the main experiment in this study. Nine separate participants (7 female) between 18 and 26 years of age (M = 20.8) were recruited to take part in an exploratory experiment designed to select optimal target sizes for the main experiment. These sample sizes are in line with those typically used in studies of human motor learning. All participants received a base pay of $10 per hour and could earn more by successfully hitting targets during the experiment (see below). Participants provided written informed consent, and after the conclusion of the experiment they were debriefed. The experiment was approved by the Queen’s General Research Ethics Board and complied with the Declaration of Helsinki.

#### Apparatus and stimuli

Seated participants used their dominant hand to grasp, using a precision grip, a rigid spherical knob located on a table in from of them. The knob, which was mounted on a force sensor (ATI Industrial Automation, N.C.), was positioned in the participant’s mid-sagittal plane approximately 30 cm from their body. The participant applied a horizontal force to the knob to ‘throw’ a circular “puck” (diameter 10 mm) from the start position to a target, both presented on a vertical screen via rear-projection (NEC UM-330W). To ensure the task was intuitive, the mapping between forces in the horizontal plane and the simulated force on the puck was the same as the standard mapping between mouse motion and cursor motion on a screen. Thus, a forward horizontal force applied to the knob (i.e., directly away from the participant) moved the puck directly up the screen, and a rightward horizontal force applied to the knob moved the puck to the right on the screen. The puck was modelled as a damped point mass using the following equation:

F=ma+bv

where *F* is the force applied to the puck, *m* and *b* are the simulated mass (0.010 kg) and viscosity (0.035 N/m/s), and *a* and *v* are the acceleration and the velocity. In all trials, the center of the target was located 30 cm straight above the start locations; i.e., the center of the puck at the start of the trial. Critically, during each throw, the simulated force could only be applied to the puck when the center of the puck was still within 10 cm of its start location. That is, once the center of the puck moved 10 cm from the start location, the simulated force was set to zero. The average time between the onset of puck motion (speed greater than 5 cm/s) and when the puck reached the 10 cm mark ranged between 92 and 126 ms across participants. Thus, controlling the puck involved predictive control and participants had to learn the correct impulse (force acting over time) to apply to the knob to produce an accurate movement of the puck to the target.

The target could be one of 6 sizes (208, 104, 64, 48, 38, and 32 mm radius), and these were associated with different point values (1, 2, 3, 4, 5, and 6, respectively) for successful throws, where 1 point earned the participant $0.02. All targets had a central black dot (5 mm radius). During target selection, which took place after every 5 trials, the 6 potential targets were displayed at the top of the screen, with the currently selected target being filled white and the others being filled blue. Participants used the left and right arrows on a keypad to select the target for the next 5 trials. Pressing the right arrow, for example, would cause the potential target to the right of the currently selected potential target to fill in white (and become the currently selected potential target). The currently selected potential target was also displayed in the target position, allowing participants to appreciate its size in the context of the task before committing to that target. Note that only potential targets equal to or smaller than the current target could be selected. Once the participant settled on a target choice, they could select it by pressing the down arrow key.

#### Selecting the 6 target sizes

Our goal when specifying the sizes of the 6 potential targets was to encourage participants to select all 6 targets in order to be optimal. More specifically, we wanted to choose target sizes that would make each target optimal—in terms of points earned—for an approximately equal similar number of trials during the learning phase, in which movement error (distance between the centers of the target and puck) decreased. To achieve this goal, we first initially ran a separate, exploratory experiment in which a group of 9 participants performed 400 throwing trials with a small target (10 mm radius). To encourage good performance, participants received an entry into a raffle for a $20 gift card each time they hit the target.

For all participants, movement error decreased approximately exponentially and reached asymptote within 200 trials. (This is the number of trials we then used in our main experiment.) Using the movement errors from these 200 trials, we determined the 6 target sizes (worth 1–6 points in decreasing size) so that for optimal reward each target is selected for roughly the same number of trials (200/6). To approximate this solution, for a given potential set of target sizes we calculated the number of trials participants would spend on each target so as to maximize reward. We then determined the maximum of these trial numbers across the targets. The optimization then chose the set of targets sizes so as to minimize this maximum. In effect, this approach tends to evenly distribute the trials across the six targets. This optimization was also constrained such that the overall hit rate had to be above 50% so that we would not end up specifying target sizes that would be demotivating for subjects. As will be shown below, this approach appeared to be effective in that, for all participants in the main experiment, both their actual performance and their optimal performance involved selecting all, or all but one, of the targets.

#### Procedure

For the first 5 throwing trials, the largest potential target (208 mm radius) was fixed as the target. After these first 5 trials, and after every subsequent block of 5 trials, the participant could select the target of their choosing using a keypad (see above). All participants performed a total of 200 throwing trials and thus made 39 target selections. We enforced two rules on target selection. First, participants could not select a larger target size than the one currently selected. For example, if a participant on the second block selected the 104 mm radius target, they could not select the 208 mm radius target on the third block, or any subsequent block. We applied this rule to constrain the target decision space we would have to assess when evaluating the optimality of participants’ target selections. [Note that without this rule, the number of possible target selection sequences (6^39^ = 2.2x10^30^) would be extremely high. With this rule, this number (1.09x10^6^) was computationally tractable]. Second, we enforced the largest target over the first 5 trials in order to give participants experience with the task before they could begin selecting a target.

At the beginning of each trial, the target and puck (located at the start position) appeared on the screen. Whenever the participant was ready, they applied forces to the joystick in an attempt to throw the puck onto the target. Participants could view the puck as it moved towards the target and came to a stop. A throw was deemed to be successful when any part of the puck, after it had come to rest, was within 1 mm of any part of the target. [Note that we added this 1 mm margin to avoid situations in which the participant falsely believed that a near-hit was a hit, which could result in frustration]. When participants hit the target, they received positive feedback in the form of an audible tone (1000 Hz, 50 ms) and the target circle momentarily turned white. In addition, text was displayed on the screen indicating the number of points earned on that trial (zero in the case of a miss), along with the average number of points per trial earned thus far.

#### Data analysis

To quantify task performance, we computed movement error as the resultant (x, y) distance between the final position of the puck and the center of the target (data available in S1 Data). To assess learning curves, we used the Curve Fitting Toolbox in MATLAB. Repeated measures ANOVA and paired t-tests were used to assess learning using an alpha level of 0.05.

#### Models

We denote the six targets *i* = 1 … 6 sorted by decreasing radius *r*_*i*_. If successfully acquired on the *k*^*th*^ trial, the *i*^*th*^ target is rewarded with *i* points. We denote a participant’s absolute movement error on the trial by *e*_*k*_.

In the lead-lag model, at each decision point (trials 5*j*, where *j* = 1, 2 …. 39) the model considers the total points, *V*, that would have been acquired for each target smaller or equal to the current target *i* for the previous *lag* and future *lead* trials:

$$V(i,j,lag,lead)=\sum_{k=max\;(1,5j-lag)}^{min\;(5j+lead,200)}iH[r_{i}-e_{k}]$$

where *i* is (by definition) the value of the target and *H* is the heaviside function that determines whether a trial is rewarded (1) or not (0):

H[x]={0,x<01,x≥0

The target selected at decision *j*, *q*_j_* is given by:

$$q_{j}^{*}=\text{arg}\;\;\underset{q\geq i}{\text{max}}\;V(q,j,lag,lead)$$

For the local optimal model, we chose the values of *lag* and *lead* for each participant that maximized the total reward that could be achieved over the 200 trials.

For the lead-lag participant fitting, we chose the values of *lag* and *lead* so as to minimize the sum of squared differences between the target number selected by the model and that selected by the participant, summed over the 39 choices. For robustness we only considered models where the sum of lead and lag was at least 5. For the lag and lead models, the value of *lead* and *lag* were set to zero, respectively, during the optimization.

For the global optimal model, we considered all possible valid (i.e. decreasing in size) sequences of targets over the 39 choices to find the target sequence that maximized the total reward that could be achieved.

In all of our models, we used participants’ actual performance to calculate the reward that would be achieved for different possible target sizes. Previous non-adaptation studies have examined expected reward by calculating bias and variance and then integrating the distribution of movement errors inside the target to estimate the probability of a reward [e.g. 40]. However, applying such a method to our two-dimensional learning task would require estimating the current (i.e., time-varying) two-dimensional bias and 3-parameter covariance matrix (as the task is asymmetric). Calculating such means and covariance matrices reliably requires a substantial amount of data, which is difficult during learning. In our task, the participant experienced the actual errors and therefore when considering lags, our method gives an accurate answer to the question of “would I have done better with a smaller target?”. Similarly, for the leads we take a practical approach of using the participants’ actual performance as an upper limit of what they might be able to estimate when asking ‘will I do better in the future with a smaller target?”

### Experiment 2: Visuomotor adaptation task

#### Participants

Seventeen right-handed participants (9 female) between the ages of 19 and 27 years old (M = 22.17) took part in the experiment after providing written, informed consent. We used a larger sample size in this experiment because we obtained, from each participant, a small number of choices (see below), which in our experience can result in greater variability [e.g., 41]. Participants received a $10 for completing the hour-long experiment, and received an additional sum of money corresponding to $0.05 per every 100 points collected throughout the experiment. This study was approved by Queen’s University General Research Ethics Board and complied with the Declaration of Helsinki.

#### Apparatus and stimuli

Participants grasped the handle of a planar manipulandum [23] with their right hand to produce the required movements (Fig 7A). The position of the handle was recorded at 1000 Hz. Visual stimuli, including a cursor controlled by the handle (filled orange circle, radius 5 mm), a start position (filled white circle, radius 5 mm), and a target (empty white circle, radius 10 mm), were projected by a computer monitor downwards onto a semi-silvered mirror that was visible to the participant and blocked vision of their hand (Fig 7A). To further prevent the participant from seeing their hand or arm, a curtain was fitted between the participant’s neck and the near side of the mirror. The height of the mirror was half way between the monitor and the center of the handle and therefore the visual stimuli appeared at the height of the center of the handle.

At the beginning of each trial, the robot applied forces to the handle so as to position it (and the cursor) at the start position. After a delay of 400 ms a target appeared at one of three pseudo randomly selected locations 100 mm from the start position and located at 0, +45, and -45 degrees relative to the start position (Fig 7B). Participants were told to initiate a single continuous movement, out to the target and back to the start position, as soon as the target appeared. If reaction time was greater than 350 ms, the participant received no points for that trial. This reaction time criteria was implemented to limit the likelihood that participants would engage cognitive strategies (e.g., mental rotation) when the visuomotor rotation was implemented [24,25,42,43]. An auditory cue sounded 500 ms after their handle left the start position and participants were instructed that the handle should arrive back at the start position in synchrony with this cue.

Participants were instructed not to make on-line corrections if the direction of the cursor did not match the direction of the target. However, to ensure that participants did not make corrections, once the cursor reached 30 mm away from the start position, a ‘force channel’ was implemented in line with the vector joining the start position and the current position of the cursor (Fig 7B). This channel constrained the handle to straight-line motion by applying force perpendicular to the direction of the channel if the handle deviated from the center of the channel. (The channel force was simulated as a damped spring with stiffness 3500 N/m and damping 2 Ns/m.).

Participants received substantial feedback on their motor performance during each trial. When the handle reached 100 mm from the start position (i.e., the distance to the target), a ‘copy’ of the cursor was displayed on the screen at that location and remained there for the rest of the trial. (If the cursor did not reach the 100 mm mark, the position of the cursor at the turn around point was extrapolated to the 100 mm mark and displayed.) Furthermore, at the end of the out-and-back movement, text was displayed indicating if the trial was a “Fault” (reaction time > 350 ms) or, if the reaction time criterion was satisfied, whether the trial was a “hit” (where any part of the cursor overlapped with any part of the target during the movement) or a “miss”. Finally, at the start of each phase of the experiment, a grid of empty white circles representing the trials to be performed—with 6 rows and N columns where 6 x N was the number of trials—was displayed and shown throughout the phase (see Fig 7C–7E). The circle corresponding to a given trial changed colour at the end of each movement, with green, red, and yellow indicating the trial was a hit, a miss, or a fault, respectively. (Note that green circles are shown as blue in the figure.) This grid of circles provided participants with a visual representation of the number of trials to be performed and also enabled participants to monitor their performance throughout the phase.

#### Procedure

After performing a series of baseline trials (see below), the 45° visuomotor rotation was implemented and the participant completed an initial learning phase of 30 trials (see Fig 7C). After these 30 trials, participants selected a reward schedule for each of three different harvesting horizons: a short (24 trials), medium (150 trials), and long (216 trials) horizon. Participants were informed that, following reward schedule selection, they would be randomly assigned to one of three ‘horizon groups’ and, depending on the group, be asked to complete an additional 24, 150, or 216 trials with the reward schedule they had selected for that horizon.

To select reward schedules, participants used a visual slider (Fig 7D) that they could control by moving the robot handle (see below). In addition to the slider, the grid of 30 coloured circles from the end of the initial learning phase was displayed along with a set of either 24, 150, or 216 unfilled circles, representing the number of trials associated with one of the three horizons. The 30 coloured circles provided participants with a visual representation of their initial performance and the unfilled circles provided participants with a visual sense of the number of trials to be performance for each horizon. Note that the order in which the different horizons were presented was randomized.

Participants selected a reward schedule by moving the cursor over the slider and depressing a button on the top of the vBOT handle to ‘grasp’ the slider. By moving the handle to the left or right participants could select a reward schedule. Once the participant was satisfied with the schedule, they confirmed their selection by releasing the button and moving the cursor over to a text box with “OK” on it and depressing the button again. The number of points for a hit and the number of points for a miss always added up to 100. The slider was initially set in the middle to 80:20 (hit:miss) and participants could adjust the slider by 1 point increments to as low as 60:40 or as high as 100:0.

After the reward schedules were selected for each learning horizon the experimenter informed the participant that, in fact, they had been assigned to a control condition in which they would perform the long horizon with a reward schedule of 80:20. This allowed us to obtain the same large number (216) of trials for each participant and to control for any effect that choosing a high or low reward schedule might have on performance, while still getting unbiased decisions from our participants. In this final phase of the experiment, participants performed 216 trials under a clockwise 45° visuomotor rotation with a reward schedule of 80:20, after which they were debriefed and paid.

Prior to performing the initial learning phase, participants completed a series of phases designed to familiarize them with the reach task and the decision procedure. Participants first completed 30 practice trials without a visuomotor rotation and with an 80:20 (hit:miss) reward schedule, receiving the same feedback as in the initial learning phase described above. Participants then selected a reward schedule for an upcoming block of 102 baseline trials. As described above, a slider was presented along with 30 coloured circles—depicting their performance from the first 30 trials—and 102 unfilled circles. They then selected a reward schedule and performed the 102 baseline trials with 7the schedule they selected.

#### Data analysis

To assess performance, we first computed the angular error of their reaches—i.e., the angle between the vector from the start position to the cursor and the vector from the start position to the target—when the cursor was displaced 100 mm away from the start location (data available in S1 Data). If the cursor did not reach the 100 mm mark, we used the position of the cursor at its maximum displacement from the start position. A trial was classified as a hit if, at any point in the movement, any part of the cursor overlapped with any part of the target, which translated into an angular error of ≤ 5.73 degrees. Movement onset was determined as the time at which two criteria were satisfied: (1) the speed of the handle exceeded 50 mm/s and (2) the handle was displaced more than 5 mm from the start location. A trial was considered to be a fault if the reaction time from target presentation to movement onset was greater than 350 ms. Repeated measures ANOVA, paired t-tests, and correlations were used to assess the results using an alpha level of 0.05.

## Supporting information

S1 Data Matlab mat file containing the data from both experiments as well information about the content and organization of the file. (MAT)
